# Enhancing research and scholarly experiences based on students’ awareness and perception of the research-teaching nexus: A student-centred approach

**DOI:** 10.1371/journal.pone.0257799

**Published:** 2021-09-27

**Authors:** Katherine Howell

**Affiliations:** School of Medicine, University College Dublin, Belfield, Dublin, Ireland; Universita degli Studi di Palermo, ITALY

## Abstract

**Objectives:**

Research is a core competency of a modern-day doctor and evidence-based practice underpins a career in medicine. Early exposure encourages graduates to embed research in their medical career and improves graduate attributes and student experience. However, there is wide variability of research and scholarly experiences offered in medicals schools, many developed with a significant degree of pragmatism based on resources and financial and time constraints. We examined undergraduate medical students’ awareness and experience of research throughout their degree to provide recommendations for implementation and improvement of research and scholarly experiences.

**Method:**

Focus groups were conducted with medical students at all five stages of the medical degree programme. Data was coded to facilitate qualitative analysis for identification of important themes from each stage.

**Results:**

Students reported positive impacts of research on undergraduate experience, future career and society in general. Two important themes emerged from the data, the opportunity for research and timing of research experiences. Early-stage students were concerned by their lack of experience and opportunity, whereas later-stage students identified the importance of research to employability, personal development and good medical practice, but ironically suggested it should be integrated in early stages of the course due to limitations of time.

**Conclusions:**

Students provided feedback for improving research and scholarly experiences, ideally involving early exposure, a clear programme overview, with equality of access and a longitudinal approach. An emerging framework is proposed summarising the important issues identified by students and the positive impacts research experiences provide for them. These recommendations can be applied to both existing and new research programmes to provide a student-centred approach designed to augment the students’ critical analysis, inspire life-long learning, enhance the student experience and inevitably train better physicians.

## Introduction

The question of how a research-intensive university can integrate and embed research into the curriculum to enhance student learning and improve graduate attributes is a topic of immense importance. The Boyer Commission Report—Reinventing Undergraduate Education: A Blueprint for America’s Research Universities (1998) stimulated debate about the nature of an undergraduate student’s experience at a research university. The value of research in education has been further emphasised in recent Irish reports such as the Hunt report in 2011 (National Strategy for Higher Education to 2030—Report of the Strategy Group). This report highlighted the intimate relationship between research and teaching, and strongly encouraged the integration of research-led teaching in Irish universities at both undergraduate and postgraduate levels.

This research-teaching nexus is particularly relevant in professions such as medicine, where evidence-based practice is essential for enhancing quality of patient care [1–3], however, a diminishing clinical scientist cohort interested in pursuing a career in academic medicine has been observed [4, 5]. The clinical scientist is widely viewed as playing a critical role in medical research [6]. Consequently, this disquieting situation has prompted the implementation of a number of initiatives including the development of a specific Academic Track scheme for medical internships in Ireland, which began in 2017 (Health Services Executive National Doctors Training and Planning Unit). This programme enables medical interns to undertake a fully supported research project with protected time in the areas of medical education, clinical research or healthcare leadership and management, to encourage an increase in clinical scientist numbers.

Although the new academic intern programme has not been fully evaluated, a review of late stage undergraduate medical students in another Irish university, revealed their significant concerns that lack of prior undergraduate research may hinder their ability to be competitive in this programme [7] and over half of students did not think their application would be successful. The impact of early research opportunities during undergraduate medical training strongly encourages doctors to pursue a career embedded in research [8, 9]. Furthermore, exposure of undergraduate students to research opportunities has been suggested to enhance effective student engagement [10] and encourage deeper learning [11]. Immersing students in a research-intensive setting improves disciplinary learning, and inculcates both discipline-specific and more generic research skills in graduates. These extensive skills are key for enhancing employability and for the ability to adapt to complexity and rapid change in modern knowledge-based economies.

Research is currently not compulsory for medical licensure, although universities should encourage students to engage in scholarship throughout their degree programme. Consequently, most medical schools are choosing to implement a range of research and scholarship components into their curriculum [12, 13]. Although some elements of these scholarship or research programmes are consistent across medical schools, the specific format, content and delivery appear unique to each institution with limited cohesion at a national or global level. Components may be compulsory or optional, delivered in self-contained units of varying length, at different stages of the degree or in some cases longitudinally throughout the curriculum [12, 13]. The establishment of such programmes within medical schools, is likely based on a range of pragmatic considerations such as resources, availability of mentors and time constraints, rather than a thorough evaluation or understanding of whether they genuinely meet the needs of students, whether they have a tangible impact on career pathways, or whether they ultimately enhance patient care [12–14]. Proposals for implementation and developing longitudinal scholarly experience projects have concentrated on the logistical difficulties and practical considerations rather than necessarily the needs of the students [14] and most programmes have not been appropriately evaluated to assess the impact they have [15].

Despite the consensus of the value of embedding research and scholarship into education, there is limited information from specific evaluation of Research-Teaching linkages in the medical curriculum, Given that delivery of these scholarly experiences varies enormously between institutions, there is little direct evidence evaluating the impact of implementing such diverse approaches [15, 16]. Therefore a thorough understanding of the needs of the students is an important consideration when planning to implement successful programmes with tangible long-term benefits.

In this study, the student perspective is evaluated in University College Dublin (UCD) a large research-intensive university, which has defined a commitment to student-focused, ‘research-led’ education in a community based on strong research-intensive disciplines. UCD Medical School provides a 6-year undergraduate medicine programme with an intake of approximately 240 students per year, including up to 70 affiliated with Penang Medical College (PMC). The programme also includes other international students (E.U. and non-E.U.), who complete the full 6-year programme in UCD, and may remain in Ireland for subsequent employment and training. The undergraduate 6-year course includes five stages; Stage 5 incorporates the final two years of clinical training in the UCD network of teaching hospitals. UCD medical school also offers a 4-year Graduate Entry Medicine (GEM) course with over 120 students in each of the 4 stages, bringing the total number of full-time medical students to over 2000.

There is no compulsory substantial research project embedded in the undergraduate medical programme, however medical students can take an optional 8-week research elective module in the summer trimester at any stage, known as Summer Student Research Awards (SSRA). These research experiences can be taken as a module for 5 credits, or simply for audit, meaning the students complete the module in addition to their normal credits. Elective modules are available to students in stage 1–4 of the undergraduate degree programme and approximately one third of undergraduate students complete this module at some stage in their undergraduate degree. A wide variety of projects are offered, including laboratory-based and hospital-based research projects, community-based projects with patient groups or charities, biomedical engineering or veterinary projects and clinical audits or observerships. A selection of the projects are carried out abroad in other institutions, and these are often competitively attained through rigorous selection processes. This programme broadly offers a significant degree of flexibility for students who choose to participate, and fundamental aspects will be similar to programmes offered within other medical schools.

In order to ensure such experiences are effective for students, it is important to understand the medical students’ perspective on the research-teaching nexus. The development of students’ awareness and perception of research throughout the medical degree is also unknown. Identifying opportunities and barriers, and defining examples of best practice, will allow us to tailor our approach to maximise the benefits for medical education.

The aim of this study was therefore to evaluate these important issues in a cohort of undergraduate medical students in UCD, to provide insight and considerations for the development of integrated research and scholarship programmes in medical schools at national and international levels.

## Methods

### Study design

All students registered to the undergraduate medicine programme from Stage 1 to Stage 5 were eligible to participate in the study. The UCD Human Research Ethics Committee granted ethical approval for the study and permission to access students was confirmed from Head of School, Dean of Medicine (Ref# LS-17-106-Howell).

An email was sent to all undergraduate medical students explaining the aims of the project, and informing students that focus groups would be carried out for each stage during the semester. For early pre-clinical stages, a brief overview of the aims of the project was explained to the class at the start of a lecture and the students volunteered to attend the focus group immediately after the lecture, with refreshments provided. In the later clinical stages of the degree, where students are based in the teaching hospitals, students were emailed and requested to voluntarily attend a focus group by specialty coordinators. The focus groups were subsequently carried out in the teaching hospitals. Five focus groups, one per stage, were facilitated by an independent research assistant and limited to 10 students per focus group (n = 7 to 10 per focus group).

### Methodological rationale and study procedure

Focus groups are a methodological approach utilising group discussion to gather data from a number of people simultaneously. Although not without limitations, they are a particularly useful tool to collect data from a representative selection of a population to identify group attitudes and experiences [17, 18]. A central characteristic of focus groups is that rather than inviting individual responses for each question, they capitalise on the interaction and communication between participants to facilitate an understanding not just of the opinions of participants, but also how those opinions were formed. Focus groups thus encourage participation and interaction, and consequently provide rich content, otherwise difficult to obtain using alternative methods [18]. In this study, the undergraduate medical cohort was considered to be a relatively homogenous population, despite potential differences between the perceived awareness and experience of early-stage and late-stage students. Participants were not pre-defined to specifically represent, for example, those who had an interest in embedding research in their future career, those who had completed research projects or students who had clinical experience and may have a different view of the relevance of research to their clinical career. Rather the random nature of participant recruitment should give a more varied set of responses, pertinent to the undergraduate medical student cohort in general and thus provide a basis for enhancing research and scholarly experiences for all students, not just those with an interest in research.

At the beginning of the focus group, students were given the focus group schedule, project information leaflet and consent form. The research assistant recorded the focus groups on two separate devices, and each focus group lasted approximately 50–60 minutes. The same questions were posed to each of the five focus groups, to ensure comparisons could be made between students’ perceptions and opinions at different stages of their medical degree. The content evolved organically through interactive discussion, meaning that not all students contributed to all questions. Rather, if the group considered their opinions had already been discussed, the research assistant moved to the next question. This approach allows the identification of emerging themes relevant to all medical students, but moreover facilitates the identification of whether these themes are more or less applicable relative to stage, gender or nationality. The focus group schedule used in this project was adapted from one used previously in a large-scale UCD fellowship project evaluating research-teaching linkages across other degree programmes [11].

#### Research questions

The study attempted to address the following broad research questions:

1. What do medical students understand about doing research in Medicine?
2. Are undergraduate medical students aware of research in the university and how has this awareness developed?
3. What research experiences do medical students have and what worked well?
4. How have research experiences, if any, impacted their learning?
5. Do they perceive research to be important in undergraduate medicine, are there sufficient opportunities and how can we improve this?

The full focus group schedule is included.

### Data analysis

Audio files from each of the five focus groups were transcribed, and the text was imported into NVivo software for qualitative analysis (QSR International). In total, approximately 5 hours of discussion was transcribed and evaluated. NVivo facilitates organisation of qualitative data in an advanced format that permits cross- referencing, queries and visualisation of data to identify patterns and themes. Students remained anonymous throughout the focus group, however identified themselves by number prior to each dialogue.

Thematic analysis is a method designed to identify and analyse patterns or themes which emerge from qualitative data [19] using the principles defined by Morse (2015) [20]. Each focus group’s transcribed file was coded for thematic analysis by both the author and research assistant independently. Each of the five focus groups was analysed within NVivo as a separate file, allowing identification of comments relative to stage. Each broad question formed a ‘parent node’, and the answers coded within specific ‘child nodes’ according to similar recurring themes. For example, identification of how students were aware of research carried out in UCD (parent node) revealed broad themes such as the ‘built environment’, ‘information from lecturers’, or ‘school emails’, with each of these categories forming a separate child node within the parent node.

Following analysis, each node included a list of linked comments, recognisable by stage. Student answers could be categorised in more than one node depending on the content of the comments. Following the initial analysis, the data was re-evaluated to combine or condense similar nodes or re-categorise if appropriate. Following the second analysis, each node was reviewed to ensure consistency of responses. Recurring themes evident throughout the focus group also emerged during the initial coding process. These nodes were defined and amalgamated during the second analysis phase. The analysis was integrated by incorporating illustrative examples of extracts from the data with the analytical narrative of the coded responses.

Data was thus examined for recurring themes within broad questions and qualitative data was expressed as the number of responses or where appropriate as percentage of total answers in each parent node. NVivo facilitates analysis of responses across stages so that any changes in students’ awareness or perception as they progressed through their degree could be identified. Differences between stages were analysed by performing matrix coding using the nodes as the matrix item and stage as the attribute. Following analysis of the focus groups, the dimensions of research-teaching linkages perceived by the student to be important were identified.

## Results

### Demographic characteristics

Students participating in the study were all recruited voluntarily and randomly across each of the five stages of undergraduate medicine. Each focus group had 7–10 participants and included 21 men (50%) and 21 women (50%) (Table 1). Penang Medical School (PMC) students, who are awarded a UCD degree but undertake a 5-year degree with their final 2.5 years of clinical training in Malaysia, accounted for four of the nine Stage 2 students but were not represented in the other stages. Students taking part in the focus groups were further categorised based on their nationality. Approximately two thirds (69%) of participants were Irish, 2 students (5%) were from the E.U. namely France and the remaining students represented 7 other countries including Malaysia, Canada, USA, Singapore, Nigeria, Botswana and Australia. This represents the multicultural nature of the course, the university and Ireland in general.

**Table 1:** 

	Men	Women	Irish	E.U.	Non-E.U.	Focus group Total
Stage 1	**4**	**5**	**7**	**0**	**2**	**9**
Stage 2	**2**	**7**	**0**	**0**	**9**	**9**
Stage 3	**7**	**0**	**6**	**1**	**0**	**7**
Stage 4	**2**	**5**	**6**	**1**	**0**	**7**
Stage 5	**6**	**4**	**9**	**0**	**1**	**10**
Total	**21**	**21**	**29**	**2**	**12**	**42**

Table showing demographics of the focus group participants. Students identified as Males or Females, and either Irish, E.U., in both cases French, or Non- E.U. from Malaysia, Canada, USA, Singapore, Nigeria, Botswana and Australia. Focus group number ranged from 7–10 per group and 42 students were included in total.

### Medical students’ awareness of research

Students were firstly asked whether they were aware that research was carried out in UCD and how that awareness developed. All participants indicated they were aware that research was carried out. There were 69 instances in total where students described how the awareness of research originated, with some students providing more than one example. This awareness stemmed predominantly (30 of the 69 responses) from information imparted by educators associated with the course. Lecturers, and to a lesser extent, demonstrators (often PhD students involved in delivery of practical classes), were mentioned by students across all stages, whereas later stage students, immersed in a clinical setting, were more likely to discuss the influence that clinical tutors had on their awareness of research. Although the lecturer may not have provided sufficient information regarding the precise nature of the research carried out, it made students aware that research was ongoing in the university.

Students’ awareness of research also arose from information sent to them from the school, particularly regarding the SSRA programme (11 of the 69 responses); this peaked at Stage 3 students which corresponds to the most likely stage that undergraduates undertake an SSRA project. Stage 4 and 5 students also discussed an intercalated MSc programme option (6 responses), and the final year medical elective (6 responses), which can potentially be a research project, although this was not widely known. The built environment surrounding the students also heightened their awareness of research activity (6 responses); specifically, students discussed a biomedical research centre adjacent to the Medical School building, but felt somewhat detached from activities within. Other minor influences included information from peers, university reputation and social media.

### Medical students’ understanding of research in medicine

Students were then asked about their understanding of what it means to do research in medicine. There were 30 responses in total. Students from all stages referred to improving our understanding of medicine (one third of all responses) and working in a laboratory as examples of what medical research means to them. As the students progressed through the course, their ability to articulate a deeper understanding of what it means to do research in medicine became apparent. Early-stage students refer to medical research as something that increases our understanding of the human body, finding new cures and advancing therapeutics. However, once students have been exposed to a clinical setting, from late Stage 3 onwards, their concept of research in medicine expands to recognise the importance of evidence-based practice, and an understanding of the valuable contribution that clinicians make to medical research and society.

Stage 1, Female

“*Erm*, *I guess it’s about contributing to the field*, *erm trying to advance it*. *You know*, *clinical trials*, *looking for new drugs to cure diseases that aren’t curable*. *Trying to progress the drugs and treatments that are out there*.*”*

Stage 5, Male

“*I guess my understanding of research in medicine has come on a lot in the last year since we have lectures in hospital with the consultants we see out on the wards*, *but who also who talk about their research interests*. *I think that reinforced the idea that medicine is evidence-based and research has to play a key part in it*. *I feel like when we were book-learning in college and stuff*, *it didn’t seem… it wasn’t as tangible the link between medicine and research*, *whereas when you are in hospital you can see that much more clearly*, *especially when the people you are learning from are talking about it*. *And erm… I guess the clinicians are best placed to see where improvements could be made*. *I feel like more so in the last year than in my pre-clinical years I have a gained an understanding of the importance of research*.*”*

### Medical students’ exposure to research in their medical degree

Students were next asked to describe instances where they had learned about research, been taught about research, or had any research experiences. Responses were coded as ‘learning about others’ research’ (*research-led*), ‘learning about research’ (*research-tutored*), ‘learning by doing research’ (*research-based*) and ‘learning to do research’ (*research-orientated*) based on the framework of Healey [11].

Broadly, the undergraduate medical students perception of their experience of research was fairly limited. Early Stage 1 and 2 students in particular articulated that they had little or no research experience. Despite their awareness of research predominantly emanating from staff discussing their research, students rarely described ‘learning about others’ research’ as a research experience. Where students described their research experiences, it was associated with describing research they had carried out i.e. ‘learning by doing research’.

Interviewer:

“*Can you identify any instances where you have learned about research*, *been taught about research*, *or had any research experiences during your studies*?*”*

Stage 2: Female

“*I wouldn’t personally count lectures as like significant contact*, *so I would answer no to this question*.*”*

Stage 1: Female

“*I wouldn’t say we had research experiences*. *Erm*, *I don’t know what is available in the school of medicine as it is very far removed from us*. *Kinda what participant 5 was saying there*, *I am not sure we can have research at the moment*, *we are not really sure what is involved*. *We are not sure what we could add*, *who is involved in the research*. *As in*, *is it to lead research*, *what knowledge do you need to have*? *If it is research assistants what do they have to do*. *We don’t know how able we have to be to actually get involved in SSRA or anything*, *‘cos we don’t know what that would mean*.*”*

Stage 4: Male

“*In terms of learning about research or being taught about research*, *we have a lot of lectures across multiple modules across multiple years on research methods and statistics and epidemiology as well*. *They are not particularly practical*, *but they give you a good sort of basis in that you emerge with an awareness of what research is*, *what kind of research exists but it always seems a little bit more theoretical than any sort of practical day to day how to go about it and one thing about these modules is they never include any sort of opportunities–it’s almost like you are studying about research but they don’t seem to presume that you are ever going to be doing research rather that you have an awareness of it so when you are reading a paper you can understand the terminology*.*”*

From Stage 3 onwards, the proportion of students who discussed their personal experiences of doing research, particularly the SSRA, increased. In some cases, late-stage students had undertaken more than one SSRA project or had independently acquired research experiences outside of the university. Approximately one third of the students in the focus group had experience of research through doing an SSRA project. This correlates closely with the number of undergraduate students completing an SSRA project in the medical school.

### The impact of research experiences on students’ learning

**Fig 1:**
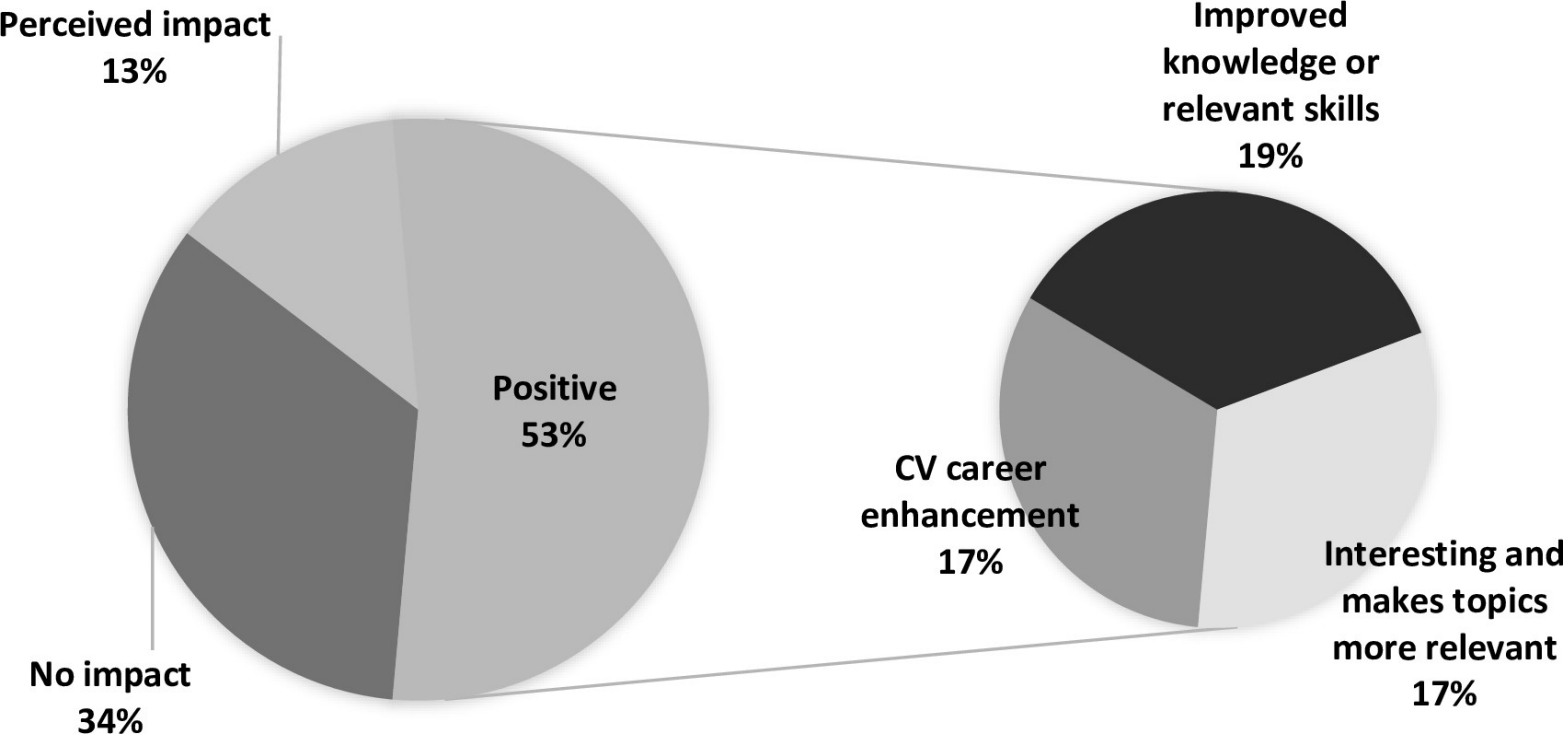
Pie of pie chart showing the impact of research on medical students. Across all 5 stages, there were 53 references or responses to the impact of research. Over half (53% of 53 responses) suggested that research had a positive impact. These positive impacts are represented in the second smaller pie chart. Approximately one third (34% of 53 responses) indicated no impact of research, predominantly because of lack of research experiences or occasionally because students did not see a role for research in their career.

The impact of research experiences on students’ learning could be categorised broadly as negative or no impact, potential/perceived impact, or positive impact (Fig 1). Approximately one third of responses (18 from 53 comments) stated that research had no impact on their learning, mostly because they had no research experience or occasionally because they did not perceive a relationship between research and learning outcomes or educational experience. In some cases, students did not see a benefit to doing research.

Stage 3 Male:

“*Erm*, *I think*, *for the vast majority of people who I would be talking to in my year would have a very practical approach to the medical degree*. *Erm*, *that I think the majority of people will be expected to work in medical practice and not in research*. *The people I hang out with generally would be focusing towards that and maybe research would be easier to avoid*, *if that is not going to be part of your career*.*”*

Some students without research experience appreciated the potential that research could have on enhancing their learning experience. Over half of responses (35 of 53 statements) described the positive effect that research had on their learning. These benefits including making subjects more relevant or enhancing their understanding or interest in a topic.

Stage 1 Female:

“*Even like the tiny bits*, *you know some lecturers would mention*, *especially in the biology ones that they are doing some research*. *It just make it more relevant*, *even regardless of what we have to do in the future it makes it easier to connect what’s going on*. *Just so*, *you know if you are just given the material and it might be*, *I don’t know*, *some material and you are told to go learn it*, *you don’t really know why you are doing*. *Whereas when they talk about the research you understand why you are being taught it*.*”*

Stage 5 Male

“*I don’t know if it’s impacted learning but more impacted your interests*. *So say like if you did a research project in a certain area*, *like*, *depending on whether you like the project or not*, *you may have an increased interest in that area*. *So it might propel you to study that topic a bit more or look into it in a bit more detail*. *But I don’t think it impacts your learning overall*.*”*

Medical students were aware of the potential impact research experiences would have on their career progression, such as enhancement of their curriculum vitae or an achievement of fulfilling an expectation. The impact on career progression was almost exclusively reported by Stage 5 students.

Stage 5 Female

“*That being said*, *I think I got a better appreciation for the fact that people within medicine are very well respected if they are researchers*, *in a lot of ways*. *So like*, *they might be clinicians by day but then*, *you know*, *researchers by night*, *but they’ll have publications and the more publications the more prestigious or like there is kind of*, *there is a respect for researchers in medicine and I think I noticed that a lot more when I was involved in the SSRA*.*”*

### Emerging themes: Opportunity and timing of research

**Fig 2:**
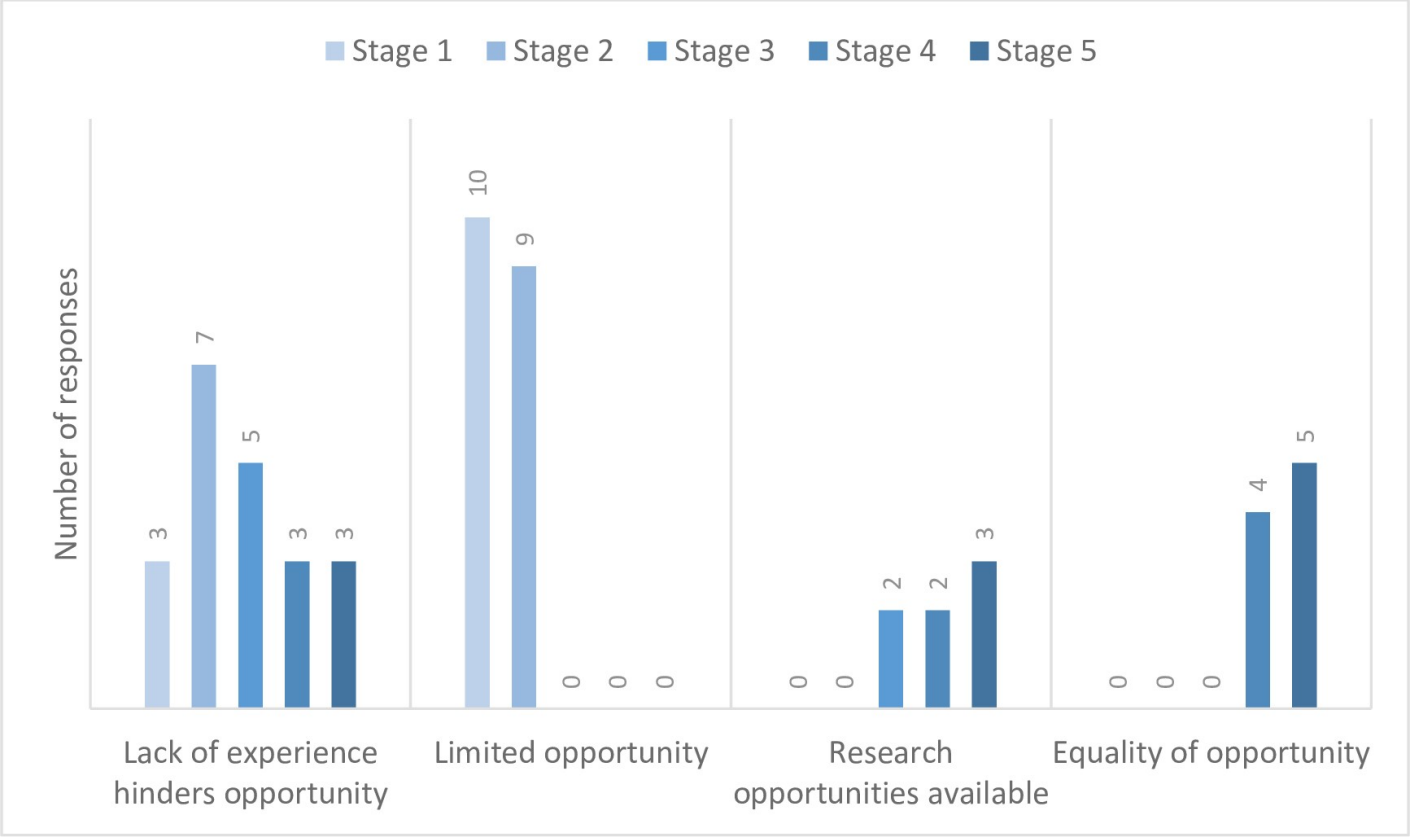
Coded responses referring to research opportunity discussed during the focus groups are represented as a bar chart showing the number of responses or references to opportunity across all 5 stages. In total, 56 responses or references to research opportunity were discussed during the focus groups. Lack of experiences being a barrier to research was discussed by students in all 5 stages. Early stage students described a lack of opportunity for research, whereas later stage students were more aware of a variety of research opportunities, however considered there was an inequality of opportunity to undertake research.

Two specific thematic areas emerged following coding of the focus group transcripts–‘opportunity for research’ and ‘timing of research’. Students reported a lack of opportunity to undertake research, particularly in early Stages 1 and 2. More importantly, students described how a lack of research experience hindered the opportunity to undertake research projects (Fig 2). This was a recurring theme throughout all stages of the programme.

Stage 1 Female:

“*I would add that*, *if applying to the SSRA because a lot of them are so specialised*, *you do need to have very specific skills if you want to do the research properly*, *so I definitely feel that is a barrier because I don’t have my research skills at this point and I feel there are very little opportunities to gain them”*

Late-stage students reported that research experiences were available, however they felt that there was an inequality of access to research opportunities, particularly if students were not available in the summer to complete an SSRA (Fig 2).

Stage 4 Female

“*[…] the SSRA projects it’s a great initiative and it has tons of projects for people to do but its I think the engagement is probably low*. *The only way I wanted to do research last summer is if I got paid for it and I ended up getting some money and so I was just very lucky that everything fell together and while I did have a great experience and I am doing research again this summer I think it was just everything falling in to place–the opportunities are sometimes hard to find*.*”*

**Fig 3:**
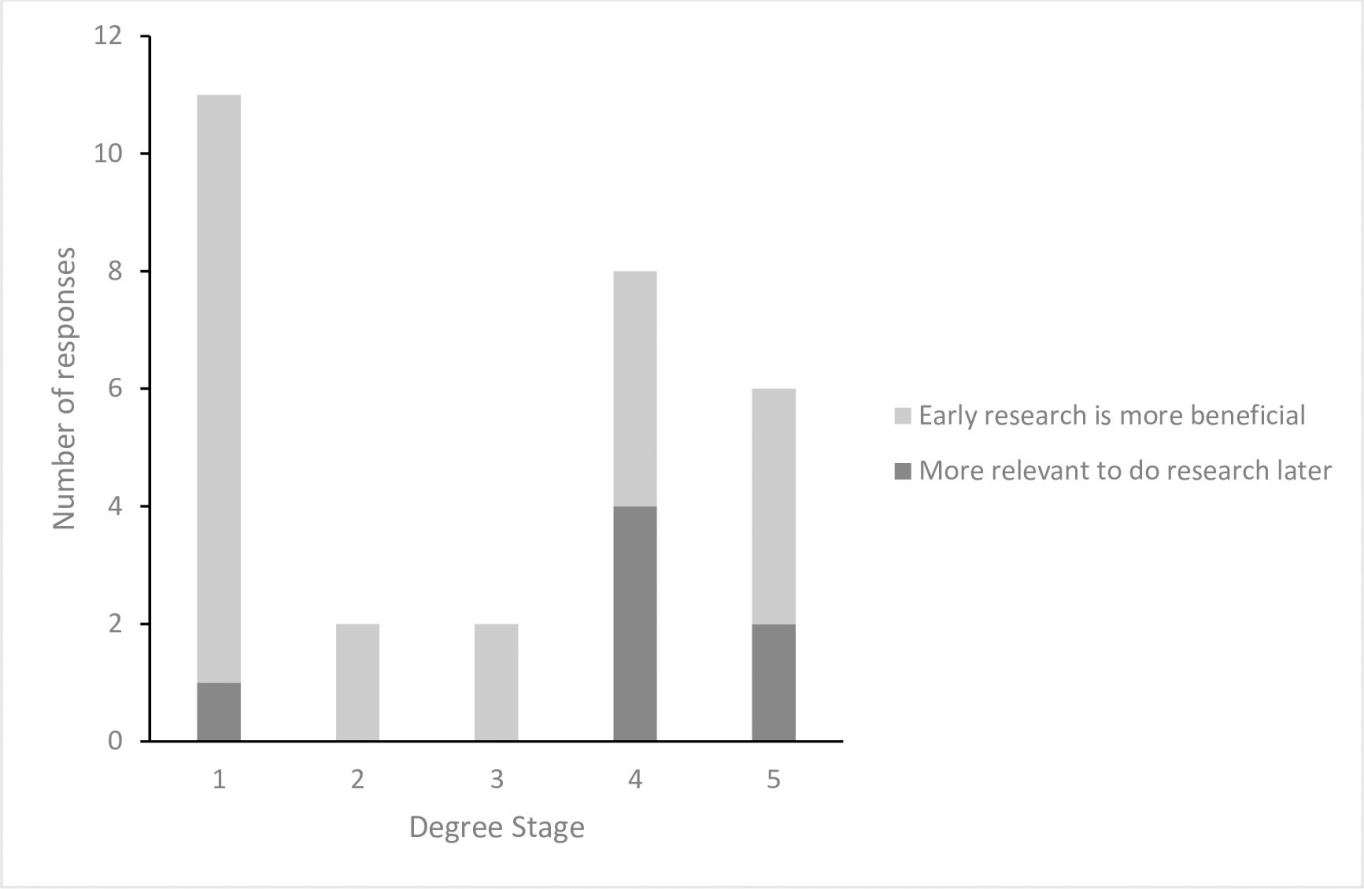
Bar chart showing the number of coded responses discussed during the focus group that were associated with the timing of research experiences. Each of the five stages of undergraduate medicine is represented individually, to facilitate an understanding of when research experiences are considered most appropriate. Students largely believed that research opportunities should be available early in the curriculum, although some later-stage students perceived that their enhanced understanding of curricular content would mean later research experiences would be more relevant.

Despite not being specifically addressed in the focus group schedule, the timing of research experiences was discussed extensively by medical students throughout the focus group session. A substantial cohort of Stage 1 students suggested that research opportunities should be available early in the course (Fig 3), however this was tempered by a consideration that lack of experience hinders their opportunity to be competitive for research projects available (Fig 3) and consequently research opportunities were more likely to occur later in their course.

Stage 1: Female

‘*At this stage I don’t want a research role as that would be a lot of responsibility but any sort of lab work would be helpful in the future because really I have no experience and I am sure everyone would agree that we have no experience in lab work and I am sure that would help us in further years in applying and getting these opportunities*. *And also I had work experience in a clinic where they were doing clinical trials and were doing research and I can definitely see how that would transfer into our professional careers in the future*. *So, it is important to start as early as possible*”

The remainder of early-stage medical students had limited interest in early research opportunities, however the majority were acutely aware that research would be necessary later in the course.

Stage 1 Male

“*I think there are very little incentives to get involved in the early stages*. *So*, *pre-med (stage 1) students wouldn’t be particularly interested in getting involved in various different types of research but I think as the years go on*, *it is not just an expectation*, *it is a necessity for us to get involved in terms of where we want to go after we graduate*.*”*

A substantial cohort of late-stage students also suggested that early opportunities for research projects would be beneficial, particularly from availability of time perspective. Elective modules are available in the earlier years, giving students the opportunity to potentially incorporate research into their curriculum. Late-stage students also acknowledged that their advanced clinical knowledge made later-stage research experiences more relevant.

Stage 4: Female

“*I think as well you need to look at the curriculum in medicine*. *I mean at the end of pre- med and with no discredit to the course you haven’t actually learned a lot about medicine and in first med you are just getting to grips with the topics and then you do pharmacology and you are getting a broader understanding of medicine so then maybe that enables you or you feel more equipped to carry out a project but then you are like*…*oh actually I have learned about this and you can relate to it better because often times as I have said before the topics for SSRA or other research projects were quite complex*, *and maybe you are like …*.*oh I understood that word but I don’t necessarily know what that means but later you are like oh I remember that from that lecture or we learned about that here”*

#### Recommendations for improvements

There was an overwhelming assertion that it is valuable to include experiences of research and/or learning about research skills in the undergraduate medical programme, however students asserted that there were insufficient research opportunities currently available. The students were subsequently asked for recommendations to rectify this situation (Table 2).

**Table 2:** Recommendations for improving research-teaching linkages.

	Stage 1	Stage 2	stage 3	stage 4	stage 5	Percentage of responses
**Do not embed research in curriculum**	**0**	**0**	**1**	**0**	**1**	**1.8**
**Early exposure to researchers, peers, clinical role models**	**2**	**0**	**1**	**6**	**3**	**10.9**
**Make research more accessible, inclusive or exciting**	**0**	**0**	**0**	**12**	**0**	**10.9**
**Improve SSRA—more structure, variety and information**	**0**	**4**	**1**	**6**	**1**	**10.9**
**Improve research information—opportunity and career importance**	**0**	**4**	**0**	**2**	**15**	**19.1**
**Embed research in curriculum**	**10**	**11**	**8**	**5**	**17**	**46.4**

The number of responses for recommendations for improvement of research-teaching linkages in undergraduate medical curriculum across all five stages. Final column shows the percentage of responses for each recommendation.

However almost half of the 110 responses to this question in the focus group recommended that research be embedded within the curriculum, either as a core component or as an elective module, and particularly around Stage 2.

Stage 4: Female

“*I think it would be better if it was included and didn’t involve giving up 2 months of your summer*, *‘cos there are loads of people who feel they have to earn money or want to travel and I think if we were able to do even 3 weeks then we have time to do other stuff as well instead of taking up the whole summer and being in UCD for another 2 months*.*”*

Stage 1 Female

“*[…]we kinda have just done science this year and so we wouldn’t be able to contribute to research*. *So then we have a disinterest*, *but maybe if the opportunity was presented to us to even observe research being done*. *Just because it will benefit us in the future*, *then if we can have that exposure we might realise how interested we are in the research*. *You know*, *it could follow from there*, *like even if we had a research elective or module where you go and watch others do research and if it was built in*.*”*

Stage 3 Male

“*[…] I see how important research is but I feel like*, *for most hospital jobs*, *you need to have done research at some stage*. *It would be great to have an introduction to it in college*. *If we were going to do research at some stage it would be good to get some introduction to research*.*”*

Stage 5: Female

“*In my mind it’s obviously a question of how much UCD is prioritising research for medical graduates to take part in*. *Because obviously it is very important to be involved in research for evidence-based medicine but*, *our only exposure to it is really through anecdotal stuff in lectures and through the SSRA and that’s like another elective five credit module*. *Whereas if there was say*, *a five or ten credit module*, *that was mandatory that focused on research*, *then we might have more of an incentive to try and get ourselves involved in research and then it would also be a UCD statement saying that we think that research is very*, *very important and so important that its worth mandatory credits*.*”*

Approximately 10% of the 110 responses requested an improvement to the SSRA programme, namely a more structured approach, more variety of projects and more information. Students also described how early exposure to researchers, peers, clinical role models was inspirational. This was linked to a request for improved research information, more research opportunity in general and specifically more information about the importance of research to a career in medicine.

Stage 4: Female

“*…we are seeing in the journal clubs here and the grand rounds these people that we could be in their position and they think research is really important*, *so if we had role models–I don’t know if you know Prof H*? *She gave the key note address at the student medical summit last year*, *just talking about how to integrate research into a clinical career*. *I think everyone came out of that thinking like*, *oh wow yeah that’s really cool and these are the steps she took and that’s something I could definitely do if I had to go down one route or another*. *It’s something to do with having role models*.*”*

Focus group schedule.

1. Are you aware that research is conducted in UCD?
  - Tell me a little about that.
  - How did that awareness develop?
2. What is your understanding of what it means to do research in Medicine?
  - Tell me a little about that.
  - How did that understanding develop?
3. Are you aware of staff in Medicine conducting research?
  - Tell me a little about that.
  - How did that awareness develop?
  - Can you talk about what you know of their research?
  - Can you explain how that knowledge developed?
4. Can you identify any instances where you have learned about research, been taught about research, or had any research experiences, during your studies?
  - Can you outline any specific examples?
  - What worked well and what did not work so well? Why was that?
  - Would you consider that you had research experiences other than the SSRA, and if so, how well did they work?
5. I’d like you now to talk now about the ways in which your awareness and experiences of research impacted on your learning.
  - Did that change over the course of your degree? (Stage 2 onwards)
  - When did that change /those changes happen?
  - Why did that change /those changes happen?
6. Do you think that it is valuable to include experiences of research, and/or learning about research skills, in undergraduate programmes?
  - In what way?
  - Do you think that your programme has provided adequate experience of, and training in, research skills? Explain.
7. We have come to the end now of the focus group. Before we finish up, is there anything that you would like to add?

## Discussion

The intimate relationship between research and teaching is now considered to be core to the effective functioning of research-intensive universities. This is particularly important in disciplines reliant on evidence-based practice such as medicine, which benefits greatly from the valuable insight provided by clinical scientists and their unique perspective from interactions with patients. The nature of the research-teaching nexus is constantly adapting to the ever-changing landscape of the educator-student dynamic [21]. The perceptions and experiences of the academic on research-teaching linkages are well-documented [22, 23], however there are obvious disciplinary and institutional contexts.

A clear inconsistency of research opportunities offered during the medical degree persists at a global level. The development of these programmes is likely driven by an element of pragmatism, coupled with a consideration of the educational ethos of the institution. These fundamental, but potentially important differences such as duration of research experiences, extent of integration, availability, content and variety of projects, assessment, governance and stage at which they are available, generate a significant variance in programmes and consequently student experience. An ability to tailor research and teaching to maximise the benefit to students and enhance graduate attributes and outcomes relies on an understanding of the students’ perception.

This study evaluated the undergraduate medical student awareness of and exposure to research in a research-intensive university. It further examined whether research experience impacted student learning, whether current research opportunities were sufficient, identified examples of best practice and sought recommendations for improvements from students. The data was analysed across the five stages of undergraduate medicine to evaluate any changes that developed throughout the course.

The demographics of the participants reflected the multi-cultural diversity of the nature of a modern Irish medical school, including the connection with Penang Medical College (PMC) in Malaysia. Not all focus groups were an exact representation of the specific demographics of that stage. For example, Stage 2 participants were all non-E.U students, including 4 from Malaysia, who were potentially associated with PMC and therefore not represented in Stage 4 and 5 because of their return to clinical training in Penang. Stage 3 participants were all male, clearly not representative of the student cohort in that year. Overall the 42 participants were reflective of the undergraduate population at the time of the study and it is likely that a sufficient number of focus groups were performed to capture the important themes [17, 24]. It has been suggested that 3 to 6 focus groups, with a homogenous population and a semi-structured discussion guide such as the focus group schedule used in this study, will likely capture 90% of all themes, including the most important ones [24]. Striking the balance between too few and too many focus groups is always open to discussion, and retrospectively it could be argued that more focus groups in each stage, or grouping pre-clinical and clinical students may strengthen the overall quality of the data.

Whilst it is possible that students from every individual medical school may also have unique perceptions on individual aspects of the study, dependent on the specific research experiences available to them, the overall themes that emerged from the data are highly likely to be relevant to the majority of medical students. The consistency of education governed by global standards determined by the World Federation for Medical Education (WFME) suggests that students are likely to have shared perceptions and opinions. Hence data presented here may be transferable and applicable to a wider international setting.

The first question in the focus group addressed whether students were aware of research and how that awareness had developed. Although all students were aware of research ongoing in the university in general, almost 45% of the responses described how their awareness of research in Medicine developed from lecturers, clinical educators and, to a lesser extent, demonstrators, who are mostly active researcher students.

Research-intensive universities have achieved a dominant position within the third-level education system, and the impact of educating students in such an environment, despite the obvious added cost, is considered valuable to the student, researchers and institution alike. Inspired by the recommendations of the Boyer Commission on Educating Undergraduates in the Research University [25], and with the growing awareness of the benefits of incorporating research experiences into undergraduate curricula, there was an explosion of interest in this area [26]. Although there was an understanding of the link between teaching and research, not all supported the concept that they were mutually interdependent (Future of Higher Education White paper UK 2003), advancing the concept of teaching-only institutions in the UK. However, many case studies have been reported and reviews have concluded that the benefits are real and substantial [27–32], albeit when care is taken to avoid potential pitfalls [33]. This growing awareness of the positive influence on the student experience and graduate attributes has narrowed the gap between research and teaching in the academic setting, encouraging academics to attempt to incorporate their research into their lectures and creating scholarly research experience programmes such as the SSRA programme described here.

Incorporating *research-led* experiences [11] for students in this study has a positive impact on students’ awareness of the research ongoing in the university, however some students articulated a disconnect, either because these discussions of research were not assessed, or because it was not relevant to their studies. This is perhaps unsurprising given the suggestion that active involvement in research by students i.e. *research-based* experiences are the most effective form of research in terms of maximising depth of learning [11]. Moreover, despite the good intentions of staff to incorporate their research into their teaching, students did not report these circumstances of ‘learning about others’ research’ or *research-led* as a research experience.

This study also highlighted the impact of the built environment on students’ awareness of research in medicine. The presence of research centres on campus inculcates an awareness from as early as recruitment days in secondary schools, and some students iterated the positive influence this had on university choice. Surrounding the medical students in an environment of research can potentially stimulate research-mindedness, however most early-stage students in this study were unaware of the research carried out, further precipitating a sense of disconnect.

This disconnect between early-stage students and their comprehension of research was evidenced in terms of their verbalisation of understanding of what it meant to carry out research in medicine. All students appeared to understand that doing research in medicine furthered our understanding of clinical medicine and potentially contributed to improving society. However, later-stage students had a greater appreciation for the relevance, importance and clinical applicability that research served, discussing evidence-based practice and how their understanding of what research means has changed after doing research or as they progress through their course and experience how research impacts on clinical practice.

Addressing this disconnect between students and staff and research and teaching at an early stage must be priority in all research-intensive institutions. A number of models have been proposed to address these issues, however, student engagement must be at the heart of any proposals [11, 34, 35]. This is likely to involve a significant shift in how we structure and deliver the undergraduate curriculum, not just at a modular, programme or institutional level but at national and international levels.

This study also evaluated the impact that research had on students’ learning throughout their degree. Unsurprisingly, the later-stage students who were more likely to have completed a research project, recognised the impact of research on learning. Whilst some students, particularly early-stage students, had no experience of doing research, they could still articulate the potential positive impact that doing research may provide. Approximately two thirds of responses relating to this question were positive, and referred to benefits such as career enhancement and improved knowledge and skills. Of particular significance were the comments that research was simply interesting and made learning more relevant, but did not necessarily impact on learning.

It is not uncommon for students to underestimate the impact that research has on their education [36], however, it is also likely that the delivery of a coherent structured research experience, potentially embedded in the curriculum, would permit the student to reflect on their experience and evaluate the impact more cohesively. As academics, we frequently witness a transformative effect of completion of significant independent research projects on the confidence and capabilities of students. In the absence of formal reflection, it is probable that students do not appreciate or recognise this flourishing effect on their educational journey.

One of the main themes that emerged from the data was the issue of opportunity. Students across all stages, but particularly Stage 1 and 2, described a lack of opportunities for research despite the availability of a research module. Students have the opportunity to take a research elective module in the summer, the SSRA scheme, which involves an 8-week project supervised by a mentor, culminating in the submission of an abstract to the Irish Journal of Medical Sciences, and an oral presentation of the project in poster form. Each summer over one hundred national and international SSRA projects are completed, of which just over half are undertaken by undergraduate medicine students. Typically, the undergraduate medical students choose to do this module at the end of Stage 3 and approximately a third of undergraduate students would complete the module during their undergraduate course.

From the focus group analysis it was clear that students generally choose to wait until Stage 3 to complete this research project because they perceive that a lack of experience hinders their competitiveness. Students are permitted to do an SSRA every summer if they choose to, although they can only take it for 5 credits on one occasion. It was reassuring to see a few students describe completing two or more SSRAs in different areas of research, indicating a desire to pursue research within their course.

However, there was criticism of the scheme, particularly from later-stage students, who describe an inequality of opportunity for students who do not have the ability to do research in the summer, due to inexperience or financial or personal reasons. Although some of the projects, both national and international, are formally advertised, and can be applied for by any student, many projects are sought independently by students actively contacting researchers in other institutions who work in a field that is of interest to the student, or through personal contacts. This creates a somewhat *ad hoc* system of projects, which in many ways brings a unique variety to the programme. However, the lack of structure, consistent opportunity and equality is off-putting to some students.

The second theme to emerge from the data was the issue of timing of research opportunities. Although some later-stage students suggested that research was more relevant in later stages due to their superior knowledge, there was a consistent opinion across all stages that early research opportunities would be ideal. The motivation for early introduction to research was either to enhance competitiveness later in the course to overcome lack of experience, because they had more flexibility, time or less pressure in the early part of the course, or because they could take the SSRA for credits in the first three stages to contribute to the next stage GPA.

The evidence to support the benefits of incorporating research experiences into a medical curriculum is extensive [37, 38] however much of the impetus for stimulating research predominantly focussed on MD or PhD programmes rather than the undergraduate experience [9, 39]. More recently, the emphasis has somewhat shifted to research experiences for medical students throughout their course, whether these are embedded within the curriculum or as voluntary electives [8, 9, 37, 39]. A number of large-scale funded programmes such as the Medical Student Research Fellowship Programme in the U.S. [9] and Medical Student Research projects in Norway [16] and the Netherlands [40] have been introduced to engage students at this crucially influential stage of their training and try to introduce a degree of consistency in student experience.

Research experiences provide a context for students’ learning and augment the understanding of the importance of research in their future careers. The data presented here demonstrate how understanding of research in undergraduate medical students evolves based on experience, and underlines the importance of early research opportunities to maximise the progression of this research journey. However this journey must surely not only be structured in nature but also mutually beneficial for both staff and students.

Most literature in this area looks at how research can impact on teaching and student engagement rather than the impact of teaching on research [41]. However, it has been suggested that not only does research have the ability to enhance teaching, but furthermore that teaching has the potential to enrich research [23, 42] creating a dynamic relationship between academics and students. Nurturing of this important relationship has the potential to bridge the gap between research and teaching, and also staff and students, particularly by encouraging research-intensive staff to actively become involved in partnerships with students in research. A recent study by Fanghanel et al. (2016) [43] emphasised that the engagement of students is essential for the scholarship of teaching and learning, and recommended that institutions should provide sustained undergraduate research opportunities through staff-student partnership in order to develop meaningful student engagement.

### Proposal for enhancement—Considerations for optimising the impact of research experiences for medical students

**Fig 4:**
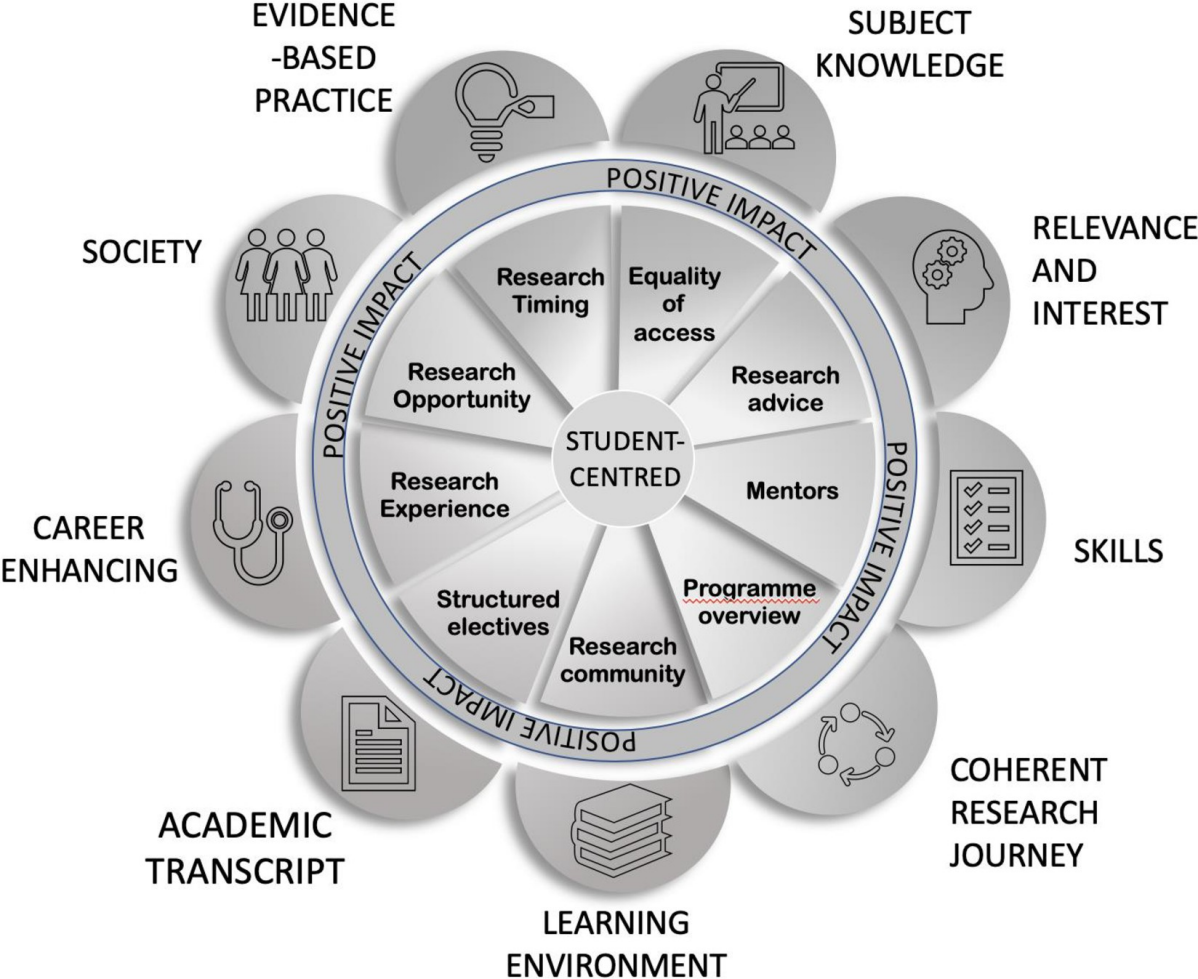
**The conceptualisation of an emerging framework places the student at the central character, identifies issues important to students (inner circle), and defines their perceived positive impacts in terms of their educational experience and future professional career (outer circle).** This framework places the student at the central character, identifies issues important to students, and defines their perceived positive impacts in terms of their educational experience and future professional career.

The recommendations of the students and important dimensions were encompassed into an emerging framework (Fig 4), which was used as a basis for suggesting enhancement to research programmes. In this study, students overwhelmingly recommended early research opportunities embedded within the course, ideally in the form of structured research electives delivered longitudinally through the course, with clear programme overview and delivered at appropriate times during the course. This would facilitate all students potentially having equal access to basic research or scholarly experiences, with the opportunity to create a significant portfolio of sequential experiences, each building on previous skills and knowledge. Students suggest that research experiences should be recorded and verified to provide a useful mechanism to substantiate students’ appropriateness for future research opportunities, suggesting a passport style portfolio may be useful. Furthermore students require valuable research techniques to enhance their CV, meaning, where possible, students should have the opportunity to complete a module on relevant research skills.

Students consistently described how naïve and inexperienced they perceive themselves to be, lacking even a basic understanding of research. Hence, an early module in the fundamentals of research, available to a large cohort of medical students, is likely to be useful in terms of enhancing student basic knowledge and experience in research. This module could include input from senior clinical scientists, acting as role models to facilitate an early understanding of the benefits of research to the medical student. Fundamental skills such as hypothesis generation, critical analysis of published articles, how to find appropriate resources to support our discussion of data or even the ability to ask pertinent questions should be incorporated into early research modules.

Subsequent modules would ideally build on this fundamental research module, potentially incorporating small research projects, exploring more detailed research topics including for example qualitative and quantitative data analysis. Given the number of students who perceive medical research to be about ‘working in a lab’, coupled with the fact that this prospect does not appeal to all students, suggests that increasing the variety of projects offered to students may be crucial to improving the student uptake. Green et al., published a compendium of examples of scholarly concentration programmes, including detailed concentration areas. Whilst biomedical sciences make up the large proportion of research projects, there are examples of some very creative non-medical projects, such as creating art programmes for patients [12].

The constraints of fulfilling academic requirements from professional bodies may provide barriers for large-scale longitudinal research experiences in the absence of significant re-structuring of existing timetables. However a number of medical schools, particularly in the U.S., have successfully incorporated longitudinal research programmes across the duration of the course culminating in the production of a dissertation. The positive impact of such programmes have been successfully evaluated [8, 12–14].

It is well-documented that the impact of students tangibly carrying out research projects is likely to be the most transformative [11], suggesting that any implementation of recommendations should, where possible, include a capstone project. This capstone project could potentially include, projects of limited duration (6–12 weeks), or more substantial such as an intercalated masters or PhD, or an M.D or clinical internship following graduation. An early opportunity to complete medicine-specific research elective modules is likely to have a significant impact on the undergraduate research journey and potentially encourage an increase in clinical scientist roles.

### Limitations of the study and future research

The use of focus groups in Healthcare and Medical education has increased exponentially over the past few decades, mostly due to the ability to gain understanding not simply *what* people think, but importantly *why* they think that way. However it is still clear that more stringent guidelines are required to help define appropriate sampling strategies, focus group number, homogenous versus heterogenous sampling balance, with the aim to maximise the methodological approach and ensure the approach is fit for purpose. In this study, it could be argued that the opinions and experiences of first year and final students may vary quite differently and therefore the undergraduate medical student cohort is not completely homogenous. Moving forward, it may be more appropriate to increase the number of focus groups from early and late- stage students, in order to analyse differences in opinions between these more homogenous groups of students and strengthen the quality of the data obtained.

The approach taken in this study was to avoid pre-conceptions during sampling, and these differences emerged naturally from the data, with early-stage (1–3) and later-stage (4–5) students expressing divergent opinions on some aspects of the discussion. This corresponded to exposure to the clinical environment, where the impact, usefulness and relevance of research could more easily be appreciated. It may also have coincided with the point at which students were more likely to have experience of independent research and scholarly experiences, giving them a more informed opinion of the value of research. However, it was also reassuring to see that although there were differences of opinion and awareness between early and later-stage students, there was also consistency across all students, particularly in their recommendations for enhancement of scholarly experiences. Furthermore, the experiences of all undergraduate students, regardless of stage, research or clinical experience were captured.

In summary, this data provides an insight into medical students perception, awareness and impact of research-teaching linkages and the opportunity to undertake scholarly activity and research as part of their medical education. Research opportunities vary considerably between medical schools, however, the goal of these experiences is to augment the students’ critical analysis, improve communication skills, inculcate a curiosity to inspire life-long learning, enhance the student experience and inevitably train better physicians. Ideally, this will increase the number of clinical scientists, a measure which will undoubtable have positive impacts on patient outcomes. Whilst pragmatic issues will inevitably dictate elements of scholarly programmes, this framework places the student at the central character, identifies issues important to students, and defines their perceived positive impacts in terms of their educational experience and future professional career.
